# Effect of total sonicated Aggregatibacter actinomycetemcomitans fragments on gingival stem/progenitor cells

**DOI:** 10.4317/medoral.22661

**Published:** 2018-09-28

**Authors:** Karim Fawzy El-Sayed, Christian Graetz, Theresa Köhnlein, Mohamed Mekhemar, Christof Dörfer

**Affiliations:** 1 Oral Medicine and Periodontology Department, Faculty of Oral and Dental Medicine, Cairo University, Egypt; 2 Clinic for Conservative Dentistry and Periodontology, School of Dental Medicine, Christian Albrechts University, Kiel, Germany

## Abstract

**Background:**

*Aggregatibacter-actinomycetemcomitans* (*A.actinomycetemcomitans*) are strongly associated with localized-aggressive-periodontitis (LAgP). The study’s aim was to test for the first time the effect of total sonicated *A.actinomycetemcomitans*-bacterial-fragments on gingival mesenchymal stem/progenitor cells’ (G-MSCs) proliferation and regenerative gene expression *in-vitro*.

**Material and Methods:**

G-MSCs were isolated, characterized, expanded and stimulated by total sonicated *A.actinomycetemcomitans*-bacterial-fragments (0 (negative-control), 15, 60, 120 and 240µg/ml; serovar-b; n=6/group). Cellular proliferation and NF-κβ (NFKB1), Alkaline Phosphatase (ALPL), Collagen-I (COL1A1), Collagen-III (COL3A1), Osteonectin (SPARC) and Osteopontin (SPP1) m-RNA expression were assessed via reverse-transcription-polymerase-chain-reaction (RT-PCR) at 24, 48 and 72 hours and CFUs-ability evaluated at twelve days.

**Results:**

G-MSCs demonstrated stem/progenitor cells’ characteristics. *A.actinomycetemcomitans*-bacterial-fragments (up to 72 hours) resulted in marked G-MSCs’ proliferation over-time (*p*<0.001) and elevated NFKB1 (*p*=0.017), COL1A1 (*p*=0.025), SPARC (*p*=0.025), decreased ALPL (p=0.017), with no significant differences for COL3A1 and SPP1 expression or stimulation times (*p*>0.05; Friedman-test). Longer-term stimulation for twelve days reduced G-MSCs’ CFUs.

**Conclusions:**

Sonicated *A.actinomycetemcomitans*-bacterial-fragments’ exert beneficial short-term effects on G-MSCs’ proliferative and non-mineralized tissue forming aptitude. Results shed new light on the importance of periodontal treatment for LAgP patients, using power driven sonic/ultrasonic devices, which, in addition to reducing the subgingival microbial load, produces cell-stimulatory *A.actinomycetemcomitans*-bacterial-fragments, with positive attributes on tissue reparative/regenerative responses of tissue resident stem/progenitor cells in their niche.

** Key words:**Ultrasonic, Aggregatibacter actinomycetemcomitans, stem cells, gingiva, Aggressive periodontitis.

## Introduction

Periodontitis, a bacterially induced inflammatory disorder, is branded by the destruction of the tooth-bearing and investing tissues; including the periodontal ligament, the alveolar bone, the cementum and the gingiva (1). Two variants of periodontal diseases categorized under Aggressive Periodontitis (AgP) (2), a localized (LAgP) and a generalized (GAgP) form, affecting 0.1%-7.6% of young healthy individuals (3), are branded by a high rate of disease progression and a tendency towards familial aggregation (4). The disease process is believed to be initiated by a microbial challenge, with a subsequent aggressive inflammatory response and periodontal tissue destruction (5). These aggressive forms mostly correlate with the presence of specific highly virulent bacterial species in the subgingival biofilm with a predominance of Gram-negative anaerobic rods (6). Although data concerning this linkage are mainly based on association studies, the Gram-negative Aggregatibacter actinomycetemcomitans (*A.actinomycetemcomitans*) species, implicated in chronic periodontitis and severe non-oral infections (seven serovars a-g) (7), is assumed, especially serovar-b, to be one of the primary etiological agents of LAgP (8,9). *A.actinomycetemcomitans* is capable of active tissue invasion (10,11), using cells as a reservoir for initial attachment, before eventually moving to non-shedding tooth surfaces, and produces a unique array of virulence factors, including the cytolethal distending toxin (Cdt) and the leukotoxin (LtxA) (12).

Reducing the *A.actinomycetemcomitans* microbial load, through mechanical removal of supra- and subgingival deposits (13) via periodontal scaling and root planning (SRP), is standardly employed during LAgP treatment and maintenance phases, to produce a biologically compatible subgingival environment for reattachment/new attachment of adjacent periodontal tissues (14). Power-driven sonic/ultrasonic instruments allow hereby an easy, fast, gentle yet thorough root surface debridement, with high efficacy and advantageous ergonomics (15-18), during surgical and non-surgical periodontal therapy. The instruments perform their action though the physical effect of their oscillating tips, increasing the convection in the otherwise stagnant or relatively slow moving subgingival fluid via acoustic micro-streaming. High-energy shock waves are alluded to be produced through cavitating gas bubbles in the liquid, which dislodge root adherent deposits, and disrupt and lyse the surrounding bacteria through the produced shear stresses (19). The produced mixture of fragmented bacterial constituents, could access the gingival connective tissue through periodontal pocket-lining inflammatory-induced micro-ulcerations and the surgical/non-surgical therapy, interacting with resident gingival mesenchymal stem/progenitor cells (G-MSCs) in their niche, possibly affecting their cellular and regenerative attributes, during the subsequent periodontal wound healing/regenerative phases.

The present study’s aim was to test for the first time the effect of the total sonicated *A.actinomycetemcomitans*-bacterial-fragments on G-MSCs’ proliferation and regenerative gene expression in vitro.

## Material and Methods

-Isolation and culture of G-MSCs

G-MSCs isolation was done as earlier described (20,21). Concisely, after attaining the patients’ informed consent (IRB:D444/10), free gingival collars attached to extracted third molars were surgically excised at the Christian-Albrechts-University-Kiel, Germany. The gingival tissue collars were de-epithelised, cut into pieces, rinsed with alpha-modification-Minimum Essential Medium Eagle (α-MEM; Sigma-Aldrich GmbH, Hamburg, Germany) augmented with antibiotics (100 µg/ml streptomycin, 100 U/ml penicillin) and 1% amphotericine (all from Biochrom AG, Berlin, Germany) and placed in dry culture flasks (Sarstedt AG, Nümbrecht, Germany) for 30 minutes to adhere to their bottoms. Subsequently, basic medium consisting of α-MEM, augmented with 15% Fetal Calf Serum (FCS; HyClone, Logan, UT, USA), 400 mmol/ml L-glutamine (Biochrom), 100 U/ml penicillin, 100 µg/ml streptomycin and 1% amphotericine was carefully added. Flasks were incubated in 5% CO2 at 37°C and cells left to grow out and the basic medium changed three times/week. After reaching 80-85% confluence, cells were detached with 0.10% trypsin-EDTA (Biochrom) and counted. Their viability was tested using Trypan Blue (Sigma-Aldrich) to be finally seeded as 30 cells/cm² (22) in basic medium in 5% CO2 at 37°C. After the first passage cells reached 80-85% confluence, they were subjected to immunomagnetic cell sorting, using anti-STRO-1 (BioLegend, San Diego, CA, USA) and anti-IgM MicroBeads (Miltenyi Biotec, Bergisch Gladbach, Germany) antibodies, according to manufacturers’ instructions (MACS; Miltenyi Biotec). Positively sorted cell fractions (G-MSCs) were seeded out to form colony-forming units (CFUs).

-Colony-forming units (CFUs) 

To assess colony-forming efficiency, G-MSCs were cultured in basic medium at a density of 1.63 cells/cm2. Aggregates of 50 or more cells were scored as colonies. On the twelfth day, a demonstrative sample of the cultures was fixed with 4% formalin, stained with 0.1% crystal violet. From the remainder of the CFUs forming G-MSCs single colonies were detached by cell scrapers (23,24) and seeded out in basic medium.

-Multilineage differentiation potential

To test for osteogenic differentiation, 2×104 second passage G-MSCs were cultured on 6-well culture plates in osteogenic inductive medium (PromoCell, Heidelberg, Germany). As controls, G-MSCs were cultured in basic medium. At day 14, cultures were stained with Alizarin-Red (Sigma-Aldrich), to mark calcified deposits. To test the adipogenic differentiation, 3×105 second passage G-MSCs were cultured on 6-well culture plates in adipogenic inductive medium (PromoCell). As a control, G-MSCs were cultured in basic medium. The presence of lipid droplets was evaluated by Oil-Red-O staining (Sigma-Aldrich). To test for chondrogenic differentiation, micro-masses of 3×104 second passage G-MSCs were incubated with chondrogenic inductive medium (PromoCell) in 6-well culture plates (Sarstedt AG, Germany). As a control, G-MSCs were cultured in basic medium. Chondrogenic differentiation was evaluated at day 35 by staining of glycosaminoglycans with Alcian-Blue and nuclear-fast-red counter staining (Sigma-Aldrich) (25). All media were renewed three times/week.

-Bacterial strains, growth conditions and fragmentation

*A.actinomycetemcomitans* serovar-b, strain Y4, DSMZ 11123 (DMSZ, Braunschweig, Germany), originally isolated from subgingival dental plaque, were grown in BD-Bacto™ Brain-Heart-Infusion (BHI; 237500, BD, Heidelberg, Germany) under microaerobic conditions for 48 hours at 37C°. From this starter culture 10 ml was taken in 1000 ml fresh BHI and cultured overnight. A sample of the late exponential-phase cultures (OD600=0.5) were Gram-stained and the rest harvested by centrifugation at 1200 g, washed twice and resuspended in PBS. Bacteria were disrupted by 20 kHz sonication in an ice bath using 30 seconds pulses separated by 30 seconds breaks (Labsonic 2000U; B.Braun Biotech, Melsungen, Germany). The preparations were centrifuged at 12000 g for 10 minutes (JA-18, Beckman Coulter, Krefeld, Germany) to remove unfragmented cells and sterile-filtrated by passage through a 0.2 µm pore size filter (Merck Millipore, Darmstadt, Germany). Bacterial CFUs-test of the filtrate was negative, confirming the absence of living bacteria. Total sonicated *A.actinomycetemcomitans*-bacterial-fragments were determined with the Micro BCA Protein Assay kit (Thermo-Fisher Scientific, Schwerte, Germany). The toxic dose (TD50) concentration was determined at 72 hours incubation of the sonicated *A.actinomycetemcomitans*-bacterial-fragments with G-MSCs, as previously described (26).

-G-MSCs stimulation

TD50 was obtained at a concentration of 240 µg/ml total sonicated *A.actinomycetemcomitans*-bacterial-fragments. Accordingly, a downward logarithmic concentration scale was prepared. 2×104 third passage G-MSCs were cultured in 6-well culture plates. After initial cell-adhesion, five experimental groups received total sonicated *A.actinomycetemcomitans*-bacterial-fragments in basic medium at concentrations (0 (negative-control), 15, 60, 120 and 240 µg/ml, n=6/group) for 24, 48 and 72 hours each.

-Cell proliferation assays

Cell proliferation and viability was determined in the five groups at 24, 48 and 72 hours using the MTT (3-[4,5-dimethylthiazol-2-yl]-2,5-diphenyl-tetrazoliumbromide) Cell Proliferation Kit-I (Roche, Mannheim, Germany). Third passage G-MSCs were seeded in 96-well cell culture plates at a density of 1×103 cell/well. After adhesion (day 0), cells were cultured and stimulated according to the group allocation described above. At 24, 48 and 72 hours, the media were replaced by no phenol-red serum-free medium (RPMI 1640, PAN-Biotech, Aidenbach, Germany). 10 μl MTT labelling reagent (final concentration 0.5 mg/ml) was added into each well and incubated for 4 hours (37°C, 5% CO2). 1 ml of the Solubilization solution was added into each well and incubated overnight (37°C, 5% CO2). The spectrophotometrical absorbance was measured using a universal microplate spectrophotometer (μQuant, BioTek Instruments, Vermont, USA) at 570 nm wavelength. Relevant cell numbers were calculated according to standard curves. Each assay was performed in triplicate and averaged.

-CFUs-assay

To assess colony-forming efficiency, third passage G-MSCs were seeded out at a density of 1.63cells/cm2. Following their adherence, cells were stimulated according to the group allocation described above and media renewed three times/week. On the twelfth day, demonstrative cultures were fixed with 4% formalin, stained with 0.1% crystal violet. Aggregates of 50 or more cells were scored as colonies, counted independently by two examiners and averaged.

-m-RNA extraction and cDNA synthesis

m-RNA extraction was performed for third passage G-MSCs of the five groups at 24, 48 and 72 hours, using the RNeasy kit (Qiagen, Hilden, Germany), according to the manufacturer’s instructions. The obtained RNA was purified using RNase-free-DNase (Promega, Mannheim, Germany), and quantified photometrically. To perform two-step RT-qPCR, complementary cDNA was synthesized from 1-13 μl of RNA (1 μg/μl) by reverse transcription (RT) kit (QuantiTect; Qiagen), according to the manufacturer’s instructions (Mastercyclergradient; Eppendorf). In a volume of 20 μl reaction mixture containing 4 pmol of each primer, 10 μl of the LightCycler Probes Master mixture (Roche) and 5 μl specimen cDNA the second PCR step was then performed. The expression of the inflammatory gene NF-κβ (NFKB1) and the regenerative transcription factors alkaline phosphatase (ALPL), type-I collagen (COL1A1), type-III collagen (COL3A1), osteonectin (SPARC) and osteopontin (SPP1) were measured using reverse transcription-polymerase chain reaction (RT-PCR, LightCycle, Roche Molecular Biochemicals, Indianapolis, IN, USA). The housekeeping gene PGK1 was used as a reference. The Real Time Ready Assays were supplied by Roche Diagnostics ( Table 1). The relative quantification of the gene expression was performed by the 2^-ΔΔCt method. All experiments were performed in triplicate and averaged.

**Table 1:**
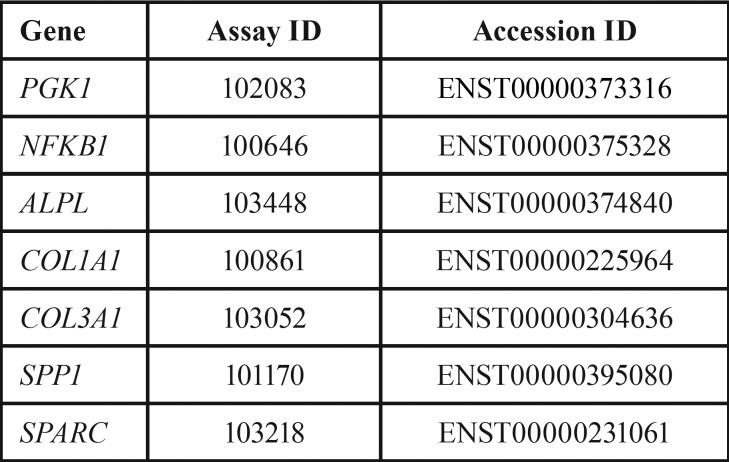
Primer names and ID used for real-time PCR (as supplied by Roche).

-Statistical analysis

Shapiro-Wilk-Test was used to test for normality of the data. Differences in cell proliferation, expression of the inflammatory gene NFKB1 and the regenerative transcription factors ALPL, COL1A1, COL3A1, SPARC and SPP1 were tested between the different experimental groups and stimulation times, using the two-way nonparametric Friedman-test. A Wilcoxon-signed-rank-test was performed for post-hoc multiple comparisons, using the SPSS software (SPSS version 20, SPSS, Chicago, IL). The level of significance was set at *p*=0.05.

## Results

-Phase contrast inverted microscopy, CFUs and bacterial staining

**Figure 1:**
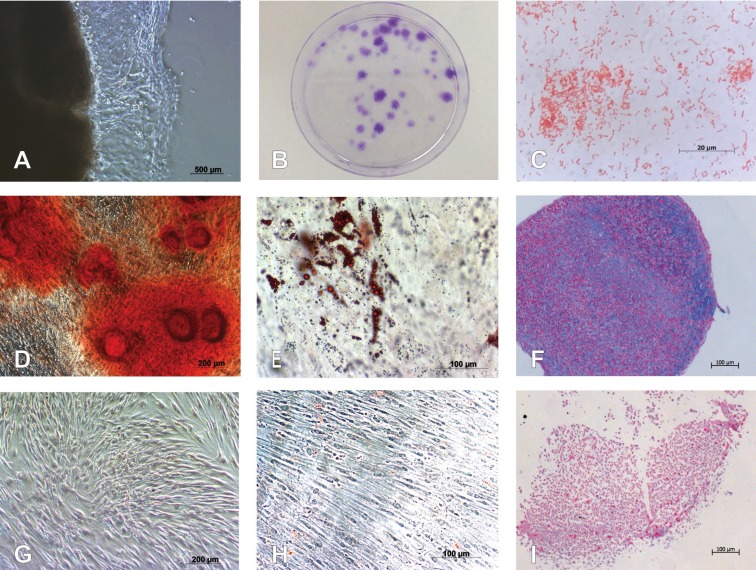
Isolation and characterization of G-MSCs. (A) Microscopic appearance of outgrowing cells from free gingival margin connective tissue. (B) Microscopic appearance of CFUs of G-MSCs stained with crystal violet. (C) Gram-staining of Aggregatibacter actinomycetemcomitans (A.actinomycetemcomitans) colonies. (D) Alizarin Red staining of G-MSCs after osteogenic induction. (E) Oil Red O staining of G-MSCs after adipogenic stimulation. (F) Alcian Blue and nuclear-fast-red counter staining of G-MSCS after chondrogenic stimulation. (G) Alizarin Red staining of G-MSCs cultured in basic medium. (H) Oil Red O staining of G-MSCs cultured in basic medium. (I) Alcian Blue and nuclear-fast-red counter staining of G-MSCs cultured in basic medium.

Following the initial adherence phase, cells grew out of the gingival soft tissue masses, forming adherent fibroblast-like clusters (Fig. 1A). Twelve days after seeding, G-MSCs showed CFUs (Fig. 1B). Late phase exponential phase of *A.actinomycetemcomitans*-bacterial colonies stained Gram-negative (Fig. 1C).

-Multilineage differentiation potential

Osteogenic differentiation of G-MSCs was demonstrated by the formation of calcified deposits labelled with Alizarin-Red, in contrast to their controls (Fig. 1D,G). Adipogenic differentiation of G-MSCs resulted in the formation of lipid droplets positively stained with Oil-Red-O, in contrast to their controls (Fig. 1E&H). Chondrogenic differentiation of G-MSCs resulted in the formation of glycosaminoglycans positively stained with Alcian-Blue and nuclear-fast-red counter staining, in contrast to their controls (Fig. 1F,I).

-G-MSCs’ proliferation and CFUs-assay

**Figure 2:**
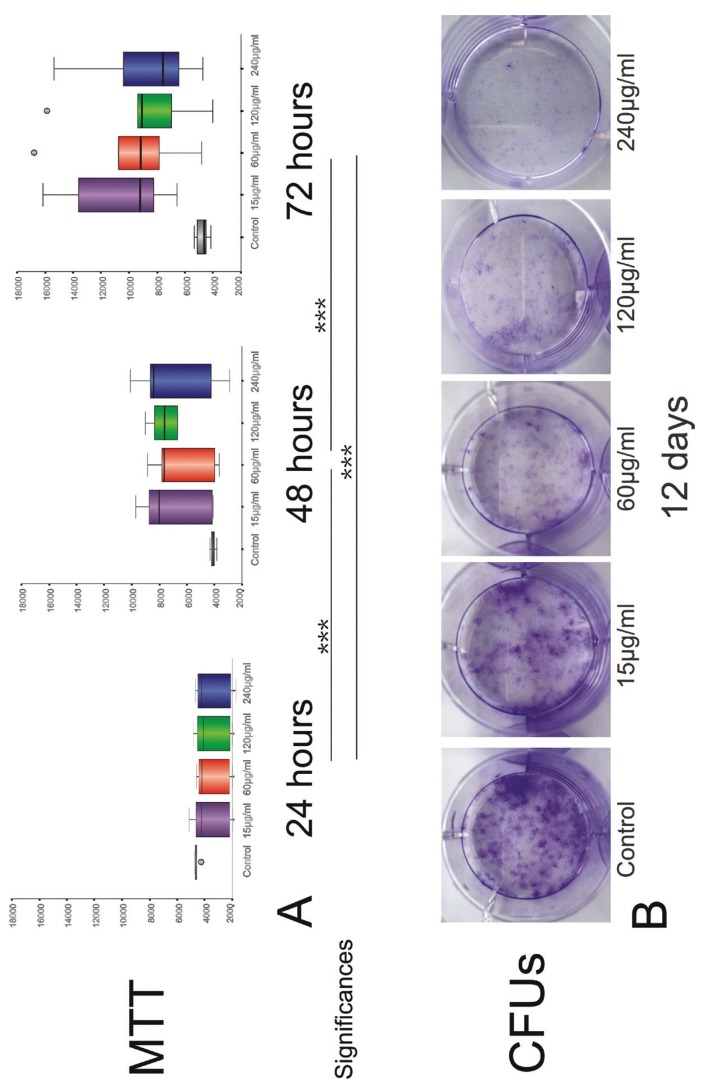
Cell proliferation and CFUs assay of G-MSCs with different concentration of total sonicated A.actinomycetemcomitans-bacterial fragments. (A) MTT results (box plots with medians and quartiles) of G-MSCs proliferation at 24, 48 and 72 hours and concentrations 0 (negative-control), 15, 60, 120 and 240 µg/ml A.actinomycetemcomitans-bacterial fragments (n=6/group). Significant differences between time-points with asterisks (***, *p*<0.001; Wilcoxon-signed-rank-test). (B) Crystal violet staining of G-MSCs CFUs for twelve days in concentrations 0 (negative-control), 15, 60, 120 and 240 µg/ml A.actinomycetemcomitans-bacterial fragments.

Total sonicated *A.actinomycetemcomitans*-bacterial-fragments stimulated groups showed generally lower G-MSCs’ proliferation compared to the negative-control group, following a 24 hours stimulation phase. At 48 and 72 hours, this pattern was reversed, with *A.actinomycetemcomitans*-bacterial-fragments stimulated G-MSCs showing higher proliferative activity. MTT results demonstrated significant increase in cell numbers over the 72 hours (*p*<0.001, Friedman-test). With G-MSCs numbers remaining almost constant in the control group at 24, 48 and 72 hours, *A.actinomycetemcomitans*-bacterial-fragments significantly activated cell proliferation between each of the time-points (*p*<0.001 Wilcoxon-signed-rank-test). The overall highest cell numbers were demonstrated with 15 µg/ml *A.actinomycetemcomitans*-bacterial-fragments stimulation at 72 hours [Median:9226.5 (Q25/Q75:7824.0/14246.3)] (Fig. 2A).

At twelve days, CFUs demonstrated a reversed pattern, with a negative effect induced by *A.actinomycetemcomitans*-bacterial-fragments on G-MSCs’ numbers. CFUs were highest in the negative-control group, and with increasing *A.actinomycetemcomitans*-bacterial fragments’ concentration, a steady reduction in CFUs was noted. On average 45.5 CFUs were observed in the negative-control group, 25 CFUs in the 15 µg/ml group, 19 CFUs in the 60 µg/ml group, 15.5 CFUs in the 120 µg/ml group and 6 CFUs in the 240 µg/ml group (Fig. 2B).

-Inflammatory and regenerative transcription factors’ expression 

**Figure 3:**
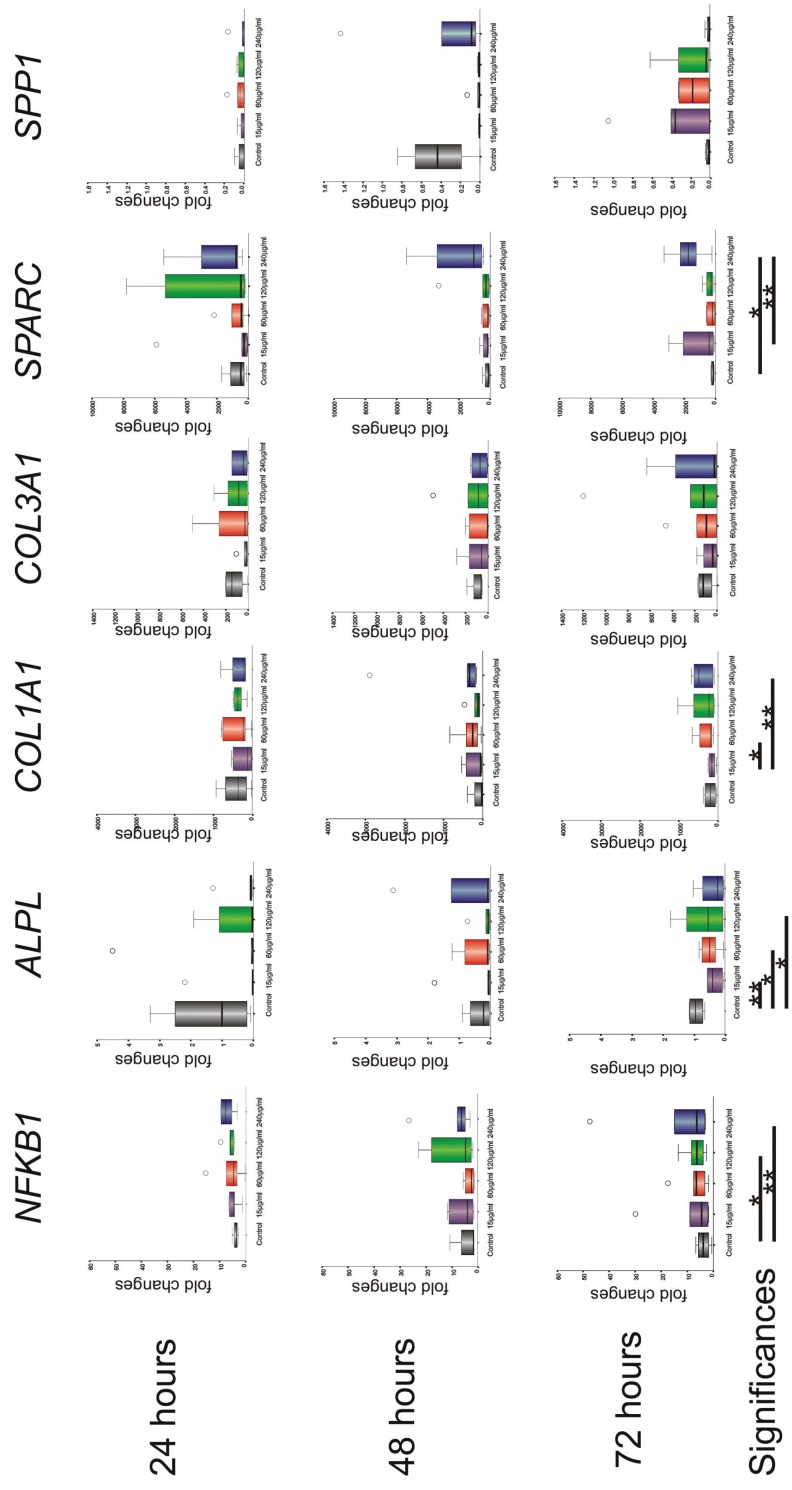
m-RNA expression (box plots with medians and quartiles) of NF-κβ (NFKB1) and the regenerative transcription factors: alkaline phosphatase (ALPL), Collagen-I (COL1A1), Collagen-III (COL3A1), Osteonectin (SPARC) and Osteopontin (SPP1) in G-MSCs at 24, 48 and 72 hours and concentrations 0 (negative-control), 15, 60, 120 and 240 µg/ml of total sonicated A.actinomycetemcomitans-bacterial fragments. Significant differences are marked with asterisks (*; *p*<0.05, **; *p*<0.01, Wilcoxon-signed-rank-test).

Challenging G-MSCs with total sonicated *A.actinomycetemcomitans*-bacterial fragments resulted in a significantly increased NFKB1 expression, with increasing *A.actinomycetemcomitans*-bacterial-fragments’ concentrations (*p*=0.017; Friedman-test). A significant increase was observed between the negative-control group and the 120 µg/ml (*p*=0.037) as well as the 240 µg/ml *A.actinomycetemcomitans*-bacterial-fragments groups (*p*=0.003; Wilcoxon-signed-rank-test). ALPL expression was significantly altered by the different *A.actinomycetemcomitans*-bacterial-fragments’ concentrations (*p*=0.017; Friedman test), with a significant reduction in expression reached between the negative-control group on one hand and 15 µg/ml (*p*=0.005), 60 µg/ml (*p*=0.041) and 120 µg/ml *A.actinomycetemcomitans*-bacterial-fragments groups (*p*=0.019; Wilcoxon-signed-rank-test) on the other. ALPL expression however demonstrated a significant increase over-time (*p*=0.035; Friedman-test), which was mostly attributable to the increased expression observed in the control group. COL1A1 expression showed a significant rise with increasing *A.actinomycetemcomitans*-bacterial-fragments’ challenge (*p*=0.025; Friedman-test), with a significance level reached between 15 µg/ml and 60 µg/ml (*p*=0.012) as well as between 15 µg/ml and 240 µg/ml *A.actinomycetemcomitans*-bacterial-fragments groups (*p*=0.003; Wilcoxon-signed-rank-test). COL3A1 expression showed no significant difference with increasing total *A.actinomycetemcomitans*-bacterial-fragments’ concentration (*p*=0.078; Friedman-test). A significantly increased SPARC expression was further observed with increasing *A.actinomycetemcomitans*-bacterial-fragments’ concentration (*p*=0.025; Friedman-test) and a significance level was reached between the negative-control group and 240 µg/ml (*p*=0.023) as well as between 15 µg/ml and 240 µg/ml *A.actinomycetemcomitans*-bacterial-fragments (*p*=0.002; Wilcoxon-signed-rank-test). SPP1 expression showed no significant differences with increasing *A.actinomycetemcomitans*-bacterial-fragments’ concentration (*p*=0.377; Friedman-test). Apart from elevated ALPL expression, no significant differences were notable over time (between 24, 28 and 72 hours) for the investigated markers (Fig. 3).

## Discussion

The rapidly-progressing LAgP, is predominantly associated with the presence of a highly virulent Gram-negative microflora, primarily *A.actinomycetemcomitans*, and an accompanying aggressive inflammatory response, culminating in excessive destruction of periodontal/tooth-supporting tissues (5). Bacterial pathogens and their associated molecules/virulence factors, detected through receptors of tissue-resident cells of the innate immune system play hereby a pivotal role, inducing the production, activation and secretion of inflammatory mediators (27), with resultant vasodilation and plasma leakage into the infected tissues, inflammatory cells’ recruitment and an imbalance in the host’s inflammatory response (28). If the *A.actinomycetemcomitans* challenge, especially *A.actinomycetemcomitans* of serovar-b, is not eliminated, through this tissue specific acute inflammatory response or/and the professional periodontal treatment, persisting in the periodontal crevice, severe tissue damage with a high rate of attachment loss and disease progression may occur (9), with a subsequent tooth loss at an early age and profound cosmetic, functional, and psychological effects (29). Multiple investigations demonstrated the effectiveness of sonic/ultrasonic devices in root surface debridement (30,31), with easier and faster root detoxification (17), disruption of the biofilm and reduction in the microbial load (16), producing a biologically compatible environment for reattachment/new attachment of adjacent periodontal tissues and their cells (15).

Tissue resident G-MSCs in the gingival lamina propria niche (20), show noteworthy periodontal tissue reparative/regenerative potential (32,33), remarkable immunomodulatory properties (21,34,35) and are more resistant to inflammatory stimuli than other oral mesenchymal stem/progenitor cells (36). During periodontal treatment of LAgP, G-MSCs may come in contact with *A.actinomycetemcomitans* and its sonicated bacterial fragments. The current study investigated the impact of different concentrations and exposure times of sonicated serovar-b *A.actinomycetemcomitans*-bacterial-fragments on G-MSCs’ proliferation and regenerative transcription factors’ expression. Sonication was standardized to exclude any human variables. Similar to previous investigations on this line of G-MSCs (21,32,33,37,38), the isolated cells showed all predefined mesenchymal stem/progenitor cells characteristics, confirming their multipotent identity, with plastic-adherence, CFUs formation and a multilineage differentiation potential into osteogenic, adipogenic and chondrogenic directions.

Earlier studies investigating single *A.actinomycetemcomitans*-associated virulence factors in isolation on human cells, including different adhesins and fimbriae (39), Cdt, LtxA and LPS (12), demonstrated their capacity to cause imbalance in the host’s inflammatory response, primarily through their ability to activate and kill inflammatory cells. Cdt inhibited the proliferation of human oral epithelial cells, but not periodontal ligament fibroblasts or cementoblasts (40). It further upregulated the expression of the Receptor activator of nuclear factor kappa-B ligand (RANKL), a crucial factor in osteoclastogenesis, in gingival fibroblasts and periodontal ligament cells (41). LtxA selectively distressed human cells of haematopoietic origin, by binding to the lymphocyte function-associated-receptor-1 (LFA-1), causing a disruption in their membrane integrity (42). Apart from triggering death of defense cells, LtxA together with LPS induced a massive pro-inflammatory response in human monocytes/macrophages with a resultant IL-1β-induced exaggerated bone loss (43,44). On immunological level, sonicated *A.actinomycetemcomitans*-bacterial-fragments were shown to induce IL-8 expression in gingival epithelial cells (45) and to exert a dose-dependent immunosuppressive effect via CD8 activation (46) and CD4/CD8 ratio disruption (47).

In contrast, the current study investigated the inflammatory-induced regenerative effect of total sonicated *A.actinomycetemcomitans*-bacterial-fragments on G-MSCs. During their tissue regenerative approaches, G-MSCs, similar to other stem/progenitor cells, pass through a primary cellular proliferative phase, followed by a consequent differentiation one, to finally repair/regenerate the injured tissues. Both cell numbers and differentiation capacity of the stem/progenitor cells are hereby pivotal to the final regenerative outcome in clinical application (48).

A sonicated *A.actinomycetemcomitans*-bacterial-fragments’ challenge resulted in an inflammatory response, branded by a significantly upregulated NF-κβ m-RNA expression in G-MSCs. The earlier reported resistance of G-MSCs to inflammatory challenges (36) was evident by the initial non-significant NF-κβ increase observed with 15 and 60 µg/ml *A.actinomycetemcomitans*-bacterial fragments’ concentrations. At higher concentrations, a substantial inflammatory response, branded by a significant upregulation in NF-κβ, was observed. Thus, it can be assumed that sonicated *A.actinomycetemcomitans*-bacterial-fragments successfully stimulated G-MSCs, inducing a predictable dose dependent inflammatory response.

In contrast to earlier studies on keratinocytes (49) and fibroblasts (50), an *A.actinomycetemcomitans*-bacterial-fragments’ challenge for up to 72 hours, further stimulated a remarkable G-MSCs’ proliferative activity, with a marked time-dependent surge in cell number, reaching up to two-fold increase compared to the negative-controls. Differences in cellular sources and their specific responses, *A.actinomycetemcomitans*-fragments’ serovars, preparation protocols, concentrations and stimulation durations could account for the observed differences. This short-term stimulation appeared beneficial in increasing G-MSCs’ quantities, to participate in the subsequent differentiation and tissue reparative/regenerative phases. In contrast, a longer-term total sonicated *A.actinomycetemcomitans*-bacterial fragments’ challenge for twelve days, exerted a detrimental effect on the G-MSCs’ cellular quantities. These results clearly highlight the specific nature of G-MSCs, their resistance to short-termed inflammatory stimuli and their positive attributes in field of regeneration under inflammatory challenge.

The respective expression of regenerative transcription factors, following the inflammatory challenge, on the other hand, showed a special pattern and points at an inflammatory-induced reparative/regenerative reaction (51). While ALPL expression showed a reduction with increasing *A.actinomycetemcomitans*-bacterial fragments’ concentration, its overall expression increased over 72 hours. Interestingly COL1A1 and SPARC expressions demonstrated the opposite trend, with a marked inflammatory-induced increase in their m-RNA expression. The observed downregulation of ALPL and the upregulation in COL1A1 with inflammation is hereby comparable to recent results observed in periodontal ligament stem cells isolated from inflamed human periodontal tissues (i-PDLSCs) (52), where inflammatory challenge similarly favored mature matrix forming genes’ expression and hindered matrix mineralization. It appears that with increasing sonicated *A.actinomycetemcomitans*-bacterial fragments’ challenge, G-MSCs enter their maturation phase into committed gingival connective tissue cells, with reduced ALPL expression, whose upregulation is characteristic for uncommitted mesenchymal stem/progenitor cells as well as differentiated osteoblasts (53). Concomitantly, an accompanying upregulation in extracellular matrix proteins’ expression of differentiating gingival connective tissue cells, including COL1A1 and SPARC (54), occurs. However due to the sonicated *A.actinomycetemcomitans*-bacterial fragments’ short stimulation time, of maximum 72 hours in the present study, it is difficult to conclude whether the downregulated ALPL expression observed should be attributed to an inflammation-induced differentiation process of G-MSCs to committed gingival extracellular matrix producing cells or to the reported effect of the *A.actinomycetemcomitans*-induced interference with cellular osteogenic mineralization phases (52,55,56).

*A.actinomycetemcomitans*-bacterial fragments’ produced via sonic/ultrasonication exert positive short-term effects on G-MSCs, primarily through stimulating their proliferative and non-mineralized tissue differentiating aptitude. Taking into consideration the physical effect of the sonic/ultrasonic oscillating tips, increasing the convection in the subgingival fluid via acoustic micro-streaming, in addition to its gingival crevice washing effect, it is reasonable to assume that the *A.actinomycetemcomitans*-bacterial fragments would remain in contact with the gingival soft tissue cells for a short time and at low concentrations following instrumentation. Thus, the outlined longer-term unfavorable effects are more remote to happen in clinical settings. Current results shed a new light on the importance of regular periodontal treatment for LAgP patients, using power driven sonic/ultrasonic devices, which, in addition to reducing the general subgingival microbial load, and in contrast to hand instruments, produce cell stimulatory *A.actinomycetemcomitans*-bacterial fragments with positive attributes on reparative/regenerative responses of tissue resident gingival stem/progenitor cells.
